# Metabolic flux analysis in adipose tissue reprogramming

**DOI:** 10.1097/IN9.0000000000000039

**Published:** 2024-03-06

**Authors:** Ashley Medina, Joanne Bruno, José O. Alemán

**Affiliations:** 1 Laboratory of Translational Obesity Research, New York University Grossman School of Medicine, New York, NY, USA; 2 Division of Endocrinology, Diabetes and Metabolism, Department of Medicine, New York University Grossman School of Medicine, New York, NY, USA

**Keywords:** obesity, inflammation, diabetes, metabolic disease, crown-like structures, adipose tissue macrophages

## Abstract

Obesity is a growing epidemic in the United States and worldwide and is associated with insulin resistance and cardiovascular disease, among other comorbidities. Understanding of the pathology that links overnutrition to these disease processes is ongoing. Adipose tissue is a heterogeneous organ comprised of multiple different cell types and it is likely that dysregulated metabolism within these cell populations disrupts both inter- and intracellular interactions and is a key driver of human disease. In recent years, metabolic flux analysis, which offers a precise quantification of metabolic pathway fluxes in biological systems, has emerged as a candidate strategy for uncovering the metabolic changes that stoke these disease processes. In this mini review, we discuss metabolic flux analysis as an experimental tool, with a specific emphasis on mass spectrometry with isotope tracing as this is the technique most frequently used for metabolic flux analysis in adipocytes. Furthermore, we examine existing literature that uses metabolic flux analysis to further our understanding of adipose tissue biology. Our group has a specific interest in understanding the role of white adipose tissue inflammation in the progression of cardiometabolic disease, as we know that in obesity the accumulation of pro-inflammatory adipose tissue macrophages is associated with significant morbidity, so we use this as a paradigm throughout our review for framing the application of these experimental techniques. However, there are many other biological applications to which they can be applied to further understanding of not only adipose tissue biology but also systemic homeostasis.

## 1. Introduction

Systemic inflammation is increasingly recognized as being an integral factor in the development of obesity-related complications ^[1]^, with white adipose tissue (WAT) inflammation specifically being an important contributor to this process ^[2]^. Though the main function of WAT is to serve as an energy depot, it is also an endocrine organ that senses changes in the body’s nutrient status and integrates these environmental cues into hormonal signals that help maintain metabolic homeostasis ^[3–6]^. WAT consists of a heterogeneous population of cells, including adipocytes, pre-adipocytes, immune cells, endothelial cells, and nerve cells ^[7]^. Adipose tissue macrophages (ATMs) make up the majority of the WAT immune cell population and have an important role in maintaining adipose tissue function ^[2]^. In lean mouse adipose tissue, ATMs exist predominantly in the M2 activation state and exhibit an anti-inflammatory phenotype ^[8–10]^. However, conditions of nutrient excess such as obesity result in dysregulation of this homeostasis via the necessary expansion of adipose tissue. This results in transition to a more pro-inflammatory phenotype within mouse adipose tissue, as evidenced by increased M1 macrophage polarization ^[8]^ and increased levels of inflammatory cytokines such as TNF-α, IL-6, MCP-1, and IL-8 ^[1]^. Human adipose tissue exhibits intermediate inflammatory macrophage phenotypes that remain under investigation ^[11,12]^.

Obesity activates a complex systemic immune response that includes the recruitment of macrophages and other immune cells to key metabolic tissues ^[13]^. A hallmark of pathologic WAT inflammation is the formation of crown-like structures (CLS), a term that refers to the encircling of necrotic adipocytes by macrophages ^[14–17]^. This histologic finding can be seen even in lean WAT and is thought to be a benign process that is essentially a means of waste removal ^[18]^. Whenever macrophage lysosomes undergo exocytosis, the apoptotic adipocytes get digested, which results in foam cell formation ^[19]^. However in obesity, adipose tissue expansion coupled with adipocyte dysfunction, local hypoxia, increased adipocyte necrosis, and increased immune cell infiltration of adipose tissue results in increased CLS formation above baseline ^[20,21]^. The function and localization of ATMs are defined by adipocyte death, as observed in adipose tissue of obese mice and humans ^[14]^ and the number of CLS has been correlated with adipose tissue inflammation, insulin resistance, cardiovascular disease, and poor cancer outcomes ^[22,23]^. Following surgery and long-term weight loss, there is an observed reduction of macrophage infiltration and modifications in chemoattractant gene expression in WAT of individuals with morbid obesity ^[24,25]^. Surgery-induced weight loss has been shown to acutely increase CLS formation with otherwise physiological transcriptional programs suggesting macrophage infiltration can also occur as part of adipose tissue reprogramming leading to remodeling ^[17]^. Taken together, these data suggest a causative role for ATM dysfunction in the development of metabolic disease with obesity.

Currently, there is little understanding of the mechanistic and quantitative sequence of events that link overnutrition to adipose tissue inflammation, CLS formation, and the subsequent resolution of CLS. Toward this end, our group developed an in vitro technique to assess and modulate CLS formation and adipose tissue inflammation within an adipose tissue on a chip model ^[26]^. There is compelling data demonstrating a “sensing” of nutritional status by adipose tissue components that results in metabolic and transcriptional changes and ultimately alters their phenotype ^[27]^. Thus, furthering our understanding of both the systemic and local metabolic changes that occur with these disease processes will prove vital in revealing the pathways that underlie the observed relationship between ATM activation and dysfunction with cardiometabolic disease and adverse health outcomes. Metabolic flux analysis (MFA) is an excellent candidate strategy to answer these experimental questions as it offers precise quantification of metabolic pathway fluxes in biological systems. These fluxes, which represent the integrated and functional state of cellular metabolism, provide insight into the biology of organisms and can ultimately aid in metabolic engineering and disease treatment efforts ^[28]^.

In this review, we provide an overview of MFA as an experimental tool and describe in further detail the main experimental technique used for MFA in adipose tissue – mass spectrometry (MS) with isotopic tracing. We focus on ^13^C-driven MFA specifically as this is the experimental technique that has been most frequently used in adipose tissue studies of this nature. Additionally, we will review the literature to date that has employed MFA to further understanding of adipose tissue biology. There has been minimal published work that has employed MFA to elucidate the role of adipose tissue inflammation in the development of metabolic disease. However we anticipate this as a future direction of the field as we become increasingly aware that dysregulated cellular metabolism can have far-reaching effects and is likely the driver of myriad disease processes.

## 2. Metabolic flux analysis – an overview

MFA is an experimental technique that calculates the rate of metabolite flow through specific native or engineered biological pathways. It is a quantitative measure of the activity of metabolic pathways and describes the balance between the production and consumption rates of target metabolites leading to the determination of the rates for multiple metabolic fluxes across a biochemical network ^[28]^. This is in contrast to traditional metabolomic techniques, which capture a snapshot of the metabolic milieu at a specific time but do not always accurately reflect metabolic activity. Distinct from other “-omics” analyses, metabolic fluxes cannot be measured directly and must be inferred from sophisticated computation models through the use of software tools such as INCA 2.0 ^[29]^ and eiFLUX ^[30]^. Researchers can use MFA to optimize metabolic pathways for the production of desired metabolites or to improve cellular function ^[31]^. Moreover, MFA is also useful for identifying metabolic changes associated with diseases, aiding in the identification of potential biomarkers or therapeutic targets ^[32–34]^.

When designing an experiment for MFA, there are three factors that must be considered based on the question being asked as well as the biological system being used: (1) whether metabolic steady state can be assumed, (2) whether stable isotope tracers will be used, and (3) if isotope tracers are being used, whether isotopic steady state can be assumed ^[35]^. Metabolic steady state occurs when a balance between the production and consumption of metabolites is achieved such that metabolite concentrations within the system of interest do not change over time. Similarly, isotopic steady state is reached when a balance between the uptake and excretion of isotopes is achieved such that isotopomer distribution does not change over time. Determining the answers to these questions will inform the type of analysis that is achievable for a given experiment (Table 1).

**Table 1:** An overview of the various types of MFA and their respective assumptions.

	Isotopic steady state	Metabolic steady state	Tracer
S-MFA	N/A	+	−
^13^C-MFA	+	+	+
^13^C-NMFA	−	+	+
DMFA	N/A	−	−
^13^C-DMFA	−	−	+

13C-DMFA, 13C-labeled dynamic metabolic flux analysis; 13C-MFA, 13C-labeled metabolic flux analysis; 13C-NMFA, 13C-labeled isotopic non-steady state metabolic flux analysis; DMFA, dynamic metabolic flux analysis; MFA, metabolic flux analysis; N/A, not applicable; S-MFA, stoichiometric metabolic flux analysis.

MFA without tracers in which metabolic steady state is assumed is known as stoichiometric MFA (S-MFA). This method assumes a stable metabolic network, which one can use to determine the flux of various intracellular metabolites. A benefit to this system is that S-MFA involves linear algebra models and depends largely on accurate measurements of extracellular metabolites, making it accessible to many researchers ^[35]^. However, S-MFA has limitations when applied to various biological systems as the number of constraints is often inadequate to quantify all the relevant intracellular metabolic pathways ^[35]^.

The use of stable isotope tracers powerfully complements metabolism research but brings about the distinction of isotope tracing in contrast to MFA. While radioactive tracers were fundamental to the discovery of biochemical pathways as we know them today, the advent of MS allowed a generation of researchers to measure isotope incorporation using stable isotopes as a proxy measure for metabolic rate. Isotope tracing can answer basic questions regarding incorporation of said label into known or new metabolic routes. In contrast, MFA requires the use of a metabolic network for calculation of metabolic rates or flux. In biological systems, ^13^C incorporation reflects carbon flux and is often associated with growth or biomass accumulation. Consequently, this stable isotope has been widely used in cancer metabolism and microbiology, where the objective of the cell in question is to replicate. Deuterium (^2^H) is used in human studies for measurement of de novo lipogenesis and biomolecule synthesis given its tolerability in the form of deuterated water (D_2_O) ^[36]^. More recently, ^18^O labeling uncovered novel metabolic pathways related to cancer and neurologic disease, but its widespread use remains limited due to the cost of gaseous ^18^O and experimental setup needs ^[37]^. Finally, doubly labeled water (^2^H_2_^18^O) is the gold standard measurement for basal human metabolic rate in free-living conditions ^[38]^.

Flux analysis at metabolic and isotopic steady state often involves the utilization of ^13^C-labeled tracers to determine fluxes and is referred to as ^13^C-labeled metabolic flux analysis (^13^C-MFA). In such studies, biological systems are typically incubated for a period of time with a specific ^13^C-labeled tracer ^[39]^. Over time, the ^13^C label gets incorporated into metabolic intermediates and products as it moves downstream through the pathway of interest. The time to reach isotopic steady state depends on a number of factors, including the specific tracer that is used, the metabolic activity level of the biological system, and which downstream metabolites are being measured ^[39]^. The main advantage of using ^13^C-MFA to measure fluxes is the fact that it incorporates information from labeled intracellular metabolites and allows quantification of intracellular fluxes that cannot be delineated from extracellular fluxes, especially at intracellular metabolic branchpoints ^[35]^. As such ^13^C-MFA allows for the examination of much more intricate metabolic network models. Nonetheless, one disadvantage of using ^13^C-MFA is that it requires more resources, both in terms of experimental and computational demands ^[35]^. While flux analysis using alternative radioactive isotopes is uncommon, multiple groups including ours are developing methodologies for implementing ^2^H and ^18^O tracing in biologically relevant scenarios ^[37,40]^.

There are also certain biological situations in which isotopic and/or metabolic steady state cannot or should not be assumed for a variety of reasons. Flux analysis at isotopic non-steady state, referred to as ^13^C-labeled isotopic non-steady state metabolic flux analysis, estimates metabolic fluxes under metabolic steady state conditions and incorporates transient ^13^C-labeling data to quantify fluxes ^[41]^. This is useful in systems where achieving isotopic steady state is not feasible due to experimental time constraints. However, the computational time is significantly higher than with other MFA techniques. Flux analysis at metabolic non-steady state or dynamic metabolic flux analysis (DMFA) is helpful in determining the acute effects of metabolic perturbations on biological systems ^[35]^. In this approach, external metabolite concentrations are measured at discrete time intervals allowing one to calculate the external rates over time and thus the average fluxes. The primary advantage of DMFA over S-MFA is that it provides insight into metabolic transients that classical MFA cannot observe, with only a moderate increase in experimental and computational demands ^[35]^. Nevertheless, because DMFA still relies on stoichiometric metabolite balancing within an assumed metabolic model, it faces the same constraints as MFA in resolving parallel pathways, cyclical pathways, and reversible reactions. Finally, ^13^C-labeled dynamic metabolic flux analysis (^13^C-DMFA) describes systems in which MFA is performed at both metabolic and isotopic non-steady state, allowing for the construction of the most detailed time-resolved metabolic flux maps in experimental conditions where both isotopic and metabolic steady state assumptions cannot be met ^[35]^. This approach is by far the most complicated and more work needs to be done to make it accessible to more researchers.

The MFA method predominantly used in metabolomics research is ^13^C-MFA. While it has notable benefits over other techniques, this approach imposes restrictions on metabolic flux, thereby requiring specific modeling assumptions. These modeling assumptions include but are not limited to: (1) the assumption that metabolic fluxes remain constant, (2) the assumption that isotopic labeling has reached steady state and remains constant throughout the experiment, (3) the assumption that there is no discrimination by cellular proteins between labeled and unlabeled molecules, (4) the assumption that the ^13^C tracers are pure, (5) the assumption that the environment inside the cell is perfectly mixed, (6) the assumption that all cells in the biological system have the same metabolic phenotype, (7) the assumption that there is no turnover of macromolecules, and (8) the assumption that the metabolic model being used accounts for all of the pertinent reactions ^[28]^. Given the complex nature of many biological systems, especially when utilizing non-model organisms, it is very difficult to meet all of these assumptions underscoring the limitations of this technique in providing accurate and biologically meaningful results. Nonetheless the quantitative measurements, ^13^C-MFA provides help in identifying key regulator steps and allows for flux comparison under different conditions, making it a valuable research tool despite these limitations.

## 3. Mass spectrometry – a tool for metabolic flux analysis

MS is an analytical technique that assists in the identification and quantification of molecules based on their mass-to-charge ratio (*m*/*z*). Ionization of a sample in either gas or liquid form enables the movement of molecules through a magnetic or electric field for detection. Depending on the energy of the ionization, fragmentation may occur. The use of MS in MFA allows for an accurate measurement of isotopic labeling patterns in metabolites, enabling one to elucidate the trajectory of a labeled starting molecule as it is metabolized in a given model system. In the tracing of isotopically labeled carbon-13-containing molecules through their metabolic pathways, fluxes of interest can be quantified. Likewise, MS is capable of measuring isotopic abundance, which aids in the calculation of pathway fluxes and labeling percentage.

The two primary methods of MS are liquid chromatography-mass spectrometry (LC-MS) and gas chromatography-mass spectrometry (GC-MS). In LC-MS, a liquid sample is separated through liquid chromatography based on its relative affinity for the mobile or stationary phase. The components are then ionized and fed into the MS. In contrast, GC-MS has a determined gas mixture acting as its mobile phase. For metabolic pathway analysis, the method used and the types of metabolites queried are highly dependent on the pathway of interest ^[41]^. GC-MS can be used when the sample of interest includes abundant amino acids, organic acids, fatty acids, and sugars following chemical derivatization ^[41]^. In addition, the data results for short-chain fatty acids are clearer for GC-MS due to their gaseous nature ^[42]^. For polar non-volatile analytes, such as sugar phosphates and acyl-CoA molecules, LC-MS is preferred as it covers a wide range of polarity and avoids thermal degradation ^[41]^.

## 4. Metabolic flux analysis in adipose tissue biology

**Figure 1.**
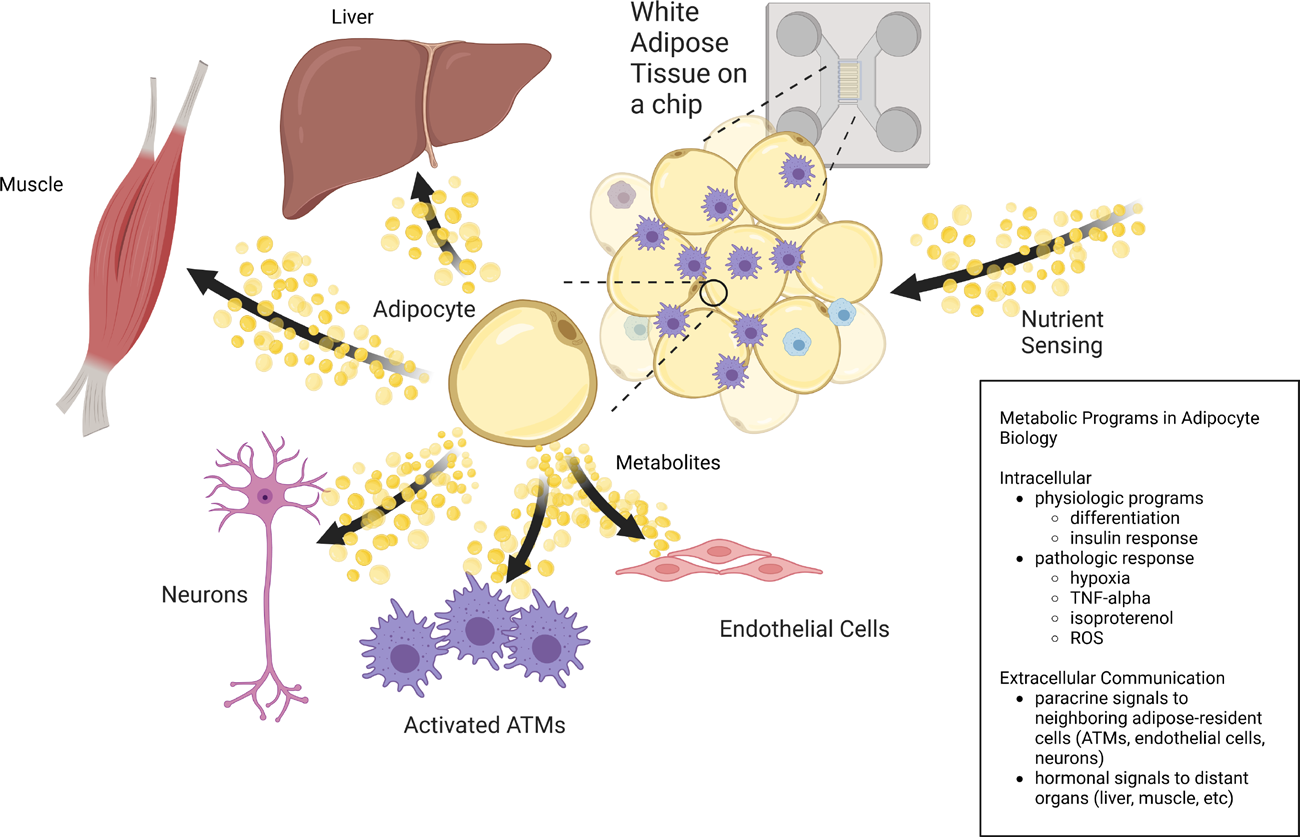
**White adipose tissue is an endocrine organ that integrates nutrient signals from the extracellular environment into hormonal and paracrine signals that help maintain metabolic homeostasis.** Metabolic flux analysis (MFA) is a useful tool for understanding the metabolic reprogramming that occurs within adipose tissue in response to these stimuli. MFA has been used to describe the metabolic shifts that occur within adipocytes as they undergo differentiation and respond to both physiologic and pathologic stimuli. Changes in intracellular metabolic flux have both local and systemic consequences as they alter the paracrine and hormonal signals that adipocytes use to communicate with neighboring cell types and target organs. While we are just starting to understand the implications of these metabolic programs and how perturbations in these systems contribute to human disease, this early data makes apparent the usefulness of MFA in making sense of these complex processes.

The observed links between nutritional status and adipose tissue function support the hypothesis that alterations in metabolic programs within adipocytes and adipose-resident cells are abettors in the processes that lead to insulin resistance and metabolic disease, and MFA is an excellent tool for uncovering which pathways might be affected. While MFA is a relatively novel enterprise within the field of adipose tissue biology, key publications have already highlighted the use of MFA in understanding the processes that drive normal adipocyte function and differentiation ^[43–45]^, the adipocyte stress response ^[46]^, the intercellular communication between adipose-resident cells ^[47]^, and the communication with adipose tissue and more distant organs ^[48]^. In this section, we will describe some of the work related to these findings as well as ruminate on the future of the field as it pertains to immunometabolism and metabolic disease summarized in Figure 1.

In normal physiology, adipose is an insulin-responsive tissue and plays a critical role in glucose and lipid metabolism ^[49]^. Recently, Quek et al ^[43]^ used ^13^C-DMFA to investigate how 3T3-L1 cultured adipocytes acutely metabolize glucose in response to insulin. They were able to identify specific segments of metabolic pathways that were differentially regulated by insulin, describing an important role for glyceraldehyde 3-phosphate dehydrogenase (GAPDH) in the flux-controlling step of glycolysis ^[43]^. The use of ^13^C-DMFA for this analysis also enabled a comparison between the speed and magnitude of pathway fluxes, showing that glycolysis operates faster than the tricarboxylic acid (TCA) cycle leading to lactate being the predominant product of glucose metabolism in these cells ^[43]^. This is consistent with previous work identifying aerobic glycolysis as a keystone of adipocyte metabolism ^[50]^. However, given the non-physiological conditions required for cell culture growth, more work needs to be done to determine the physiologic relevance of this finding in live tissues.

The adipocyte differentiation process, which culminates in the conversion of pre-adipocytes into mature adipocytes, is critical for maintaining metabolic homeostasis. Differentiation of pre-adipocytes into brown adipocytes enables an increased capacity for non-shivering thermogenesis, resulting in increased energy expenditure and reported improvements in cardiometabolic health ^[51,52]^. Few studies quantified carbon fluxes in brown adipose tissue-derived cell lines including reductive carboxylation derived from glutamine ^[53,54]^. White adipocyte depots serve as important sites for energy storage but are also responsible for the secretion of adipokines that have widespread effects on systemic metabolism ^[55]^. While the transcriptional programs that regulate the adipocyte differentiation process have been well described, the accompanying intracellular metabolic changes are poorly understood. Early studies tested the effect of uncoupling protein 1 expression on model 3T3-L1 adipocyte cell lines using external flux outputs of glucose, glycerol, and lactate to establish metabolic rewiring in the absences of stable isotope tracing ^[56–59]^. Subsequently, ^13^C-MFA data from Green et al ^[45]^ supported enhanced branched chain amino acid (BCAA) flux in differentiated adipocytes compared to pre-adipocytes and determined that inhibition of BCAA catabolism inhibited adipogenesis, indicating a functional role for this metabolic pathway in adipocyte differentiation. Recently, Oates and Antoniewicz ^[44]^ used ^13^C-MFA to analyze and quantify the flux patterns in proliferating pre-adipocytes and differentiated adipocytes. A notable shift was observed between pre-adipocytes and adipocytes in all major metabolic pathways, indicating metabolic reprogramming as a major functional component of adipose differentiation and lipogenesis ^[44,45]^. Yoon et al ^[58]^ also focused on the metabolic changes that occur during in vitro adipogenesis, also examining how these changes are influenced by the inclusion of different substrates in the culture media. To investigate the metabolic alterations during adipocyte formation, the researchers used MFA in tandem with a novel modularity algorithm. Modularity analysis is a technique used to identify modules or communities within a complex network. These modules represent groups of nodes (in this case, metabolic reactions) that are highly interconnected but have fewer connections with nodes in other modules. This study estimated intracellular fluxes using a constrained non-linear optimization method in order to minimize the error between observed and calculated exchange fluxes. This involved mass balance equations represented by a stoichiometric matrix and flux distribution vector. They showed that even after adipogenesis and adipocyte differentiation has completed, metabolic changes within the adipocytes continue to occur. These changes involve shifts in the distribution of reaction fluxes within key metabolic pathways including lipogenesis, pentose phosphate pathway, and the malate cycle. Furthermore, there was an observed correlation between the redistribution of reaction fluxes around the pyruvate node and the induction of lipogenic activity suggesting that pyruvate-related reactions play a crucial role in lipid accumulation in adipocytes. Finally, Yoon et al ^[58]^ explored the effects of different substrates within the culture media revealing that while collagen gel cultures enhanced differentiation-related metabolic activities, they inhibited pre-adipocyte proliferation again speaking to the effects of non-physiologic growth conditions potentially confounding experimental outcomes.

In addition to describing normal adipocyte physiology, MFA has also been used to further our understanding of how alterations in adipose tissue nutrient flux contribute to human disease. Local hypoxia in the setting of adipose tissue expansion has been proposed as a major inciting factor in the development of adipose tissue inflammation ^[60]^. Performing ^13^C-MFA on cultured adipocytes revealed that both short- and long-term hypoxia resulted in cellular metabolic reprogramming ^[46]^. Under hypoxia, there were derangements in energy production as well as in the metabolism of glucose, glutamine, and BCAA, and the production of odd-chain fatty acids and monounsaturated fatty acids, highlighting the susceptibility of adipocytes to changes to in their extracellular microenvironment ^[46]^. Alterations in intracellular flux patterns have consequences for neighboring cells, as demonstrated in Feng et al ^[47]^, which used MFA to identify increased adipocyte-derived lactate production as a danger signal to promote the polarization of ATMs toward a pro-inflammatory state in a mouse model of diet-induced obesity. Relatively few studies leverage adipose-on-chip technology with MFA methodologies, and we aim to use such technologies to better understand pairwise interactions between adipocytes and other cellular components such as neurons, immune cells, and endothelial cells within adipose tissue (Figure 1) ^[26]^. For example, recent work identified polyamine messengers between adipocytes and vascular cells and MFA deployed within adipose on a chip would be a candidate strategy to elucidate flux changes that govern the production of such metabolites ^[61]^. MFA also led to the discovery that enhancement of de novo lipogenesis in adipocytes has effects on whole body metabolism, in part through the action of C16:1n7-palmitoleate – an important lipokine that has systemic metabolic effects including enhanced glucose disposal, improved circulating lipid profiles, and attenuation of hepatic steatosis ^[48,62]^. While traditional metabolomics rather than MFA were used to identify this metabolite, MFA was used to investigate the tissue specific effects of palmitoleate in metabolic homeostasis and inform our understanding of its role in systemic metabolism.

While few studies have used MFA to describe metabolic networks in multiple organ systems simultaneously, work done by Silva et al ^[63]^ developed integration methods using stable isotope tracers to investigate the contribution of specific substrates to the synthesis of triglycerides, fatty acids, and glycerol in various tissues. The primary goal of the study was to understand how different substrates are utilized in the process of de novo lipogenesis and glycerol-3-phosphate synthesis within the liver, mesenteric adipose tissue, and subcutaneous adipose tissue. Using this integration method, deuterated water was used to measure de novo lipogenesis and glycerol-3-phosphate synthesis. This was combined with a ^13^C nuclear magnetic resonance method that quantifies triglycerides, fatty acids, and glycerol enrichment from a specific [U-^13^C] precursor, allowing for the estimation of this precursor’s contribution to de novo lipogenesis and glycerol-3-phosphate synthesis. By adding radiolabeled fructose and glucose to drinking water, this work described how these nutrients contributed to de novo lipogenesis and glycerol-3-phosphate synthesis in target organs; namely identifying that adipose tissue had lower rates of de novo lipogenesis and glycerol-3-phosphate than the liver, and that these tissues preferentially utilized glucose over fructose for triglyceride synthesis. Despite the progress made in this field thus far, there is still work to be done so that we can fully understand how adipose metabolic reprogramming contributes to cardiometabolic disease. Adipose tissue displays metabolic flexibility, and future research should examine how it adapts to different stimuli ^[64,65]^ as it is likely that maladaptation of these processes can be identified in disease pathology. Work from our lab is geared toward understanding if and how adipocytes differentially respond to disparate stress stimuli and how they go on to communicate this duress with neighboring cells, such as ATMs. We hypothesize that adipocyte stress induction results in metabolic reprogramming and excess secretion of a metabolite messenger that shifts ATM polarization to a pro-inflammatory phenotype. MFA will be instrumental in uncovering the nutritional shifts that occur within both the adipocyte and the ATMs during these processes with the ultimate goal being the identification of key metabolic pathways that can provide therapeutic targets for metabolic disorders such as type 2 diabetes, obesity, and cardiovascular disease. At this time, it remains challenging to interrogate metabolic flux at the single cell level, posing difficulties for working with heterogeneous cell populations either in vivo or in vitro. MS imaging is a potential solution to this as it allows for investigation of molecular spatial distribution in fixed specimens ^[66]^. This is a relatively novel technique with evolving use for biological research. It has recently been employed for quantitative mapping of fluxes within tumor microenvironments ^[67]^, the first reported instance of its use for MFA, positioning it as a promising tool for describing metabolic flux at the cellular level in a variety of contexts.

An additional goal of the field is to transition from in vitro cell culture models to in vivo systems. While technologically challenging, this is imperative for more thoroughly elucidating the complex metabolic pathways underlying human physiology – both normal and pathologic. Few in vivo studies utilizing MFA have been reported to date in part due to the complex nature of these analyses that have required the innovation of computing and analytic software in order to do so. Notably, a study by Deja et al ^[68]^ focuses on the utilization of MFA to investigate ketogenesis, which refers to the production of ketones by the liver that occurs during carbohydrate scarcity. Overcoming the challenge of accurately measuring in vivo ketone production led to the development of a dual-tracer method involving acetoacetate and beta-hydroxybutyrate tracers, enabling more precise quantification of ketone turnover while considering their interconversion. Furthermore, special attention was given to addressing the inherent instability of acetoacetate as both an analyte and a tracer. This novel MFA approach utilized Elementary Metabolite Units frameworks to implement a two-pool model of ketogenesis, providing estimates for rates of appearance, disposal, and exchange of these ketones. Ultimately this research sheds light on the intricacies of ketone metabolism in vivo, providing valuable insights into its effects on physiologic and pathologic conditions and while also serving as a rare example of how MFA can be employed to describe in vivo metabolism.

## 5. Conclusions

The application of MFA in the study of adipose tissue biology has yielded significant insights, including the identification of metabolic pathways that are fundamental to adipocyte physiology as well as those that are involved in the pathophysiology that relates adipose tissue dysfunction to human disease. The continued use and refinement of this technique will be integral in furthering understanding of the complex metabolic processes at play in adipose tissue dysfunction particularly in non-adipocyte cells including ATMs. With the increasing sophistication but also the accessibility of MFA, researchers will be better equipped to uncover the underlying mechanisms of adipose tissue dysfunction and develop targeted interventions for the prevention and treatment of metabolic disorders and adverse health outcomes.

## Author contributions

A.M. and J.B. wrote the paper; J.O.A. had primary responsibility for final content. All authors have read and approved the final manuscript.

## Conflicts of interest

A.M. and J.B. declare no conflicts of interest. J.O.A. is currently acting as a consultant for Novo Nordisk and formerly served as a chair on Novo Nordisk’s Data and Safety Monitoring Board without compensation.

## Funding

J.B. has been supported financially by NIH NHLBI institutional training grant 5T32HL098129-12. J.O.A. has been supported financially by the Doris Duke Charitable Foundation, the American Heart Association 17-SFRN33490004, and K08 DK117064 – Metabolic Flux Analysis of Obesity-Associated Inflammation in Weight Loss.
